# Leveraging Next-Generation Sequencing Application from Identity to Purity Profiling of Nucleic Acid-Based Products

**DOI:** 10.3390/pharmaceutics17010030

**Published:** 2024-12-28

**Authors:** Rucha Wadapurkar, Swarda Deo, Renuka Khanzode, Ajay Singh

**Affiliations:** Gennova Biopharmaceuticals Ltd., ITBT Park, Hinjawadi Phase 2 Rd, Hinjewadi Rajiv Gandhi Infotech Park, Hinjawadi, Pune 411057, India; rucha.wadapurkar@gennova.bio (R.W.); swarda.deo@gennova.bio (S.D.); khanzode.renuka@gmail.com (R.K.)

**Keywords:** NGS, nucleic acid, mRNA vaccine, COVID-19, sequencing

## Abstract

**Background/Objectives**: The nucleic acid-based product (NAP) portfolio is expanding continuously and provides safer curative options for many disease indications. Nucleic acid-based products offer several advantages compared to proteins and virus-based products. They represent an emerging field; thus, their quality control and regulatory landscape is evolving to ensure adequate quality and safety. Next-Generation Sequencing (NGS) is mostly recommended for NAP identity testing, and we are leveraging its application for impurity profiling. **Methods**: We proposed a workflow for the purity assessment of NAPs through short-read Illumina NGS followed by data analysis of mRNA vaccine and pDNA samples. We determined the sequence identity, DNA and RNA contamination, off-target RNA contamination, and poly-A count with the proposed workflow. **Results**: Our workflow predicted most of the critical quality controls of mRNA vaccine and plasmid DNA samples, especially focusing on the identity and the nucleotide-based impurities. Additionally, NGS data interpretation also assisted in strategic decisions for NAP manufacturing process optimizations. **Conclusions**: We recommend the adaptation of incremental NGS data by regulatory agencies to identify nucleotide-based impurities in NAPs. Perhaps NGS adaptation under cGMP compliance needs to be deliberated with the regulatory bodies, especially focusing on the methods qualification and validation part, starting from the sample collection, NGS library preparation, NGS run, and its data analysis pipeline.

## 1. Introduction

Several nucleic acid-based products (NAPs) have been approved for human usage by various regulatory agencies in the last few years, and many more are lined up [1]. These products are synthesized chemically, enzymatically, and through host propagation. The rapid and safe production of these products with adequate quantity controls, especially focusing on integrity and purity, is necessary to ensure efficacy, avoid costly delays and poor clinical outcomes, and avoid threatening regulatory approvals. Another crucial requirement is the development of methods that facilitate scalable, fast, simple, and economically high-quality NAP production. 

mRNA a kind of NAP was first discovered in the early 1960s [2]; however, its popularity and applicability have remained dormant for decades. The first ex vivo injection of mRNA into human dendritic cells in the year 2002 [3] marked a significant milestone in the field of vaccinology and immunotherapy. Researchers further explored the “mRNA platform” for various healthcare modalities that are not just limited to vaccines but also cell therapy (ex vivo or in vivo) and protein replacement therapy. In the year 2023, the Nobel Assembly at the Karolinska Institutet awarded the Nobel Prize in Physiology or Medicine to Katalin Karikó and Drew Weissman for their discoveries concerning nucleoside base modifications that were used for the development of mRNA vaccines by Moderna (Cambridge, MA, USA) and Pfizer-BioNTech (USA and Germany). 

Present techniques may not be optimal for mass manufacturing, even though producing “therapeutic”-quality mRNA is not very difficult at this time [4,5]. Most mRNA production protocols involve distinct enzymatic reactions starting from DNA templates, in vitro transcription (IVT) for mRNA, and 5′ mRNA capping, followed by purification by precipitation or column chromatography. Removing nucleic acid impurities from the desired stretch of nucleotides that are mostly represented in oligonucleotides, mRNAs, siRNAs, and plasmid DNA (pDNA) is challenging. The method of choice here is the chromatographic separation or enzymatic degradation of unwanted nucleic acids. Both methods have certain challenges; thus, the precise quantitation of nucleic acid impurities is highly recommended. The most vital parameters for a quality control method are sensitivity and coverage. The qPCR, spectroscopic methods, HPLC, and fluorescence-dye-binding spectroscopic methods are the commonly used quality control methods to estimate nucleic acid impurities [6]. However, these methods have many limitations, and there is a need for a highly sensitive technology with broader coverages. A data leak of the European Medicines Agency (EMA) database has highlighted that initial batches of Comirnaty^®^ contained unexpectedly low quantities of intact mRNA along with several truncated and modified mRNA species in the finished product [7]. Furthermore, concern about the quality of mRNA vaccines again became the topic of deliberation with the data of Kevin McKernan, showing nanogram to microgram quantities of pDNA in the mRNA doses [6,7,8]. Therefore, accurate quality assessment is paramount for the success of commercialized products in the market. 

Next-Generation Sequencing (NGS) is globally accepted nowadays for assessing mRNA vaccine quality because of its robustness and precision [9]. Further, NGS has also been instrumental in the diagnosis of genetic diseases and in paving the way for their treatment [10]. NGS is an umbrella term that includes a collection of all the post-Sanger sequencing technologies for DNA and RNA sequencing. It includes different technologies like sequencing-by-synthesis (Illumina, San Diego, CA, USA), sequencing-by-ligation, and ion semiconductor sequencing (Ion Torrent, Thermo Fisher Scientific, Waltham, MA, USA). Studies comparing different sequencing platforms (Illumina and Ion Torrent) have shown that Illumina generates uniform-sized reads from both ends of a fragment (“paired-end” R1 and R2 reads) [11,12,13]. Marginally, the evolving Oxford Nanopore sequencing technology outweighs Illumina technology in terms of read length. However, Illumina chemistry is preferred over its competitors due to its Q30 read quality. The advent of targeted or amplicon sequencing using high-throughput chemistry has become a method of choice for the characterization of NAPs. The primary advantage of using NGS is that it requires a small amount of sample and provides both depth and width for the polynucleotide chain. 

Herein, we demonstrated a quality assessment workflow for NAPs by performing short-read RNA sequencing and analysis of 14 mRNA vaccines and one plasmid DNA (pDNA) sample. We have performed the identity (in terms of sequence and poly-A count) and purity tastings (DNA and RNA contamination and off-target RNA). With the proposed workflow of NGS, we have demonstrated how accurately the quality control of mRNA can be evaluated. 

## 2. Materials and Methods

### 2.1. Illumina mRNA Sequencing

The different batches of mRNA were prepared from the linearized plasmid of pDNA-614 or pDNA-628.2 using T7 RNA polymerase as described previously [14]. pDNA-614 or pDNA-628.2 was developed in-house and contains 4 “non-structural proteins” (nsp1–4) from Venezuelan Encephalitis alpha-virus [15,16,17,18] along with untranslated regions (UTRs) at the 5′ and 3′ end, a kanamycin selection marker, pMB1 plasmid origin, and the spike protein of a SARS-CoV-2 variant flanked by ApaI and SacII restriction sites. pDNA-614 encodes for the spike protein of the B.1 variant encoding the D614G mutant form. pDNA-628.2 encodes for the spike protein of the BA.1 omicron variant. The nucleotide sequences of the antigenic portion of 614 and 628.2 are deposited into the DDBJ database (LC769019 and LC769018). The mRNA synthesized from pDNA-614 is labeled as mRNA-614 followed by their batch numbers (E1–E10); similarly, the mRNA from pDNA-628.2 is named mRNA-628.2 following the batch numbers (E1–E4). The mRNA quality was checked by denaturing agarose gel electrophoresis with an Agilent 5200 Fragment analyzer (Agilent Technologies Inc., Santa Clara, CA, USA, cat no. DNF-471-0500) and quantified using a Quant-it^TM^ RiboGreen RNA Assay Kit and RiboGreen RNA Reagent (ThermoFisher Scientific Inc., Waltham, MA, USA, cat no. R11490). For sequencing, all the mRNA samples were normalized to a concentration of 100–200 ng/µL and fragmented using FPF (Fragment, Prime, Finish) mix following the manufacturer’s protocol (TruSeq Stranded mRNA Library Prep (96 Samples), cat no. 20020595). The first strand of cDNA was synthesized using random hexamer primers, FSA (First Strand Synthesis Act D) mix, and SuperScript II Reverse Transcriptase following the manufacturer’s protocol. Next, the second strand was synthesized using SMM (Second Strand Marking Master) mix. Before ligating the Illumina adapters, the 3′ ends were adenylated using ATL (A-Tailing) mix. The indexing adapters were then ligated using the ligation (LIG) mix and Illumina-TruSeq RNA UD Indexes (96 indexes, 96 samples) (Illumina, cat no. 20022371). The adapter-ligated fragments were then purified using AMPure XP beads and amplified using polymerase chain reaction (PCR) using PCR Master Mix (PMM) and PCR Primer Cocktail (PPC). 

The individual libraries from all the mRNA batches were quantified using a Qubit™ High Sensitivity dsDNA Quantification Assay Kit (ThermoFisher Scientific Inc., cat no. Q32854), and fragment distribution was checked on an Agilent 5200 Fragment analyzer with a dsDNA DNF-935 Reagent Kit (1–1500 bp) (Agilent Technologies Inc., cat no. DNF-935-K1000). Next, each library was then normalized and pooled at 4 nM concentration. The pooled library was denatured and diluted as per the manufacturer’s protocol and sequenced on the MiniSeq sequencer (Illumina Inc., San Diego, CA, USA).

### 2.2. Illumina Plasmid DNA Sequencing

Plasmid DNA (pDNA-628.2) was isolated from the *E. coli* research bank using the Qiagen miniprep kit (QIAGEN Plasmid Mini Kit, Redwood City, CA, USA, cat no. 12125). The plasmid quality was checked by agarose gel electrophoresis and quantified using a Quant-it^TM^ PicoGreen DNA Assay Kit and PicoGreen DNA Reagent (ThermoFisher Scientific Inc., cat no. P11496). The sample was normalized to a concentration of 100–500 ng/µL, fragmented, and tagged with Illumina adapter sequences (Illumina DNA Prep 24 Samples, cat no. 20060060 and Nextera DNA CD Indexes 24 Indexes, 24 Samples, 20018707). Briefly, Bead-Linked Transposomes (BLTs) and Tagment Buffer 1 (TB1) were used to tagment DNA following the manufacturer’s protocol. The adapter-tagged DNA on the BLTs was washed with Tagment Stop Buffer (TSB) and Tagment Wash Buffer (TWB). Next, the tagmented DNA was PCR amplified using Nextera^TM^ DNA CD Indexes and Enhanced PCR Mix (EPM), and finally purified using Illumina Purification Beads (IPBs). The library quality was checked on the Agilent 5200 Fragment analyzer with a dsDNA DNF-935 Reagent Kit (1–1500 bp) (Agilent Technologies Inc., cat no. DNF-935-K1000). The library was normalized to a concentration of 1.2–1.3 pM and sequenced on the MiniSeq sequencer (Illumina Inc.).

### 2.3. Bioinformatics Analysis of mRNA Sequencing

The raw sequencing data of all the mRNA samples were pre-processed using the Fastqc [19] tool and trimmed using Trimmomatic version 0.39 [20] software with a Q30 threshold. The pre-processed reads of mRNA were mapped with Ref-2 for their respective variants (614 with Ref-2_614 and 628.2 with Ref-2_628.2) using the Bowtie2 tool. The resultant SAM files were sorted and indexed using SAMtools version 1.13. This step generated sorted and indexed BAM and other mapping files. The bam files were visualized using Integrated Genomics Viewer (IGV) [21] to check the sequence identity and poly-A length. 

Next, the unmapped reads were extracted and mapped on the kanamycin and plasmid backbone region (Ref-3_614 or Ref-3_628.2) using SAMtools view -S -b -f 4 option, and alignment statistics were generated. Later, the unmapped reads from this analysis were converted to a FASTA file using SAMtools FASTA and mapped against the *E. coli* DH5α reference genome using the Basic Local Alignment Search Tool (BLAST) of NCBI. The reads representing the off-target RNA (antisense RNA) were checked. These antisense reads originated from the reverse strands [11]. The reads originated from the forward strand, detected as second in a pair of a forward strand, with SAMtools commands -b -f 128 -F 16, and first in a pair of the reverse strand, -b -f 80 or reverse strand; detected as second in a pair if they mapped to the reverse strand, with SAMtools commands -b -f 144, and first in a pair if they mapped to the forward strand, -b -f 64 -F 16 [11]. 

### 2.4. Bioinformatic Analysis of Plasmid DNA Sequencing

Similarly, raw sequencing reads of plasmid DNA were pre-processed using Fastqc and Trimmomatic-0.39 and aligned with Ref-1. The resultant SAM files were sorted and indexed, leading to the generation of bam files. The unmapped reads from the bam files were extracted using SAMtools and mapped against the *E. coli* DH5α reference genome by BLAST. 

## 3. Results and Discussion

Approximately 1 billion doses of COVID-19 mRNA vaccines had been administered globally by the end of 2023. The intensification of the mRNA manufacturing process and at-scale optimization were the keys to achieving this milestone. The analytical or quality control methods are continuously evolving to ensure mRNA vaccine identity, purity, and safety. We at Gennova Biopharmaceuticals Ltd., India, have indigenously developed and received the emergency use authorization for GEMCOVAC^®^-19 and GEMCOVAC^®^-OM. Both vaccines are based on a self-amplifying mRNA platform and are presented as lyophilized thermostable formulations. GEMCOVAC^®^-19 targets the D614G mutant of the spike protein of SARS-CoV-2, and GEMCOVAC^®^-OM targets the spike protein of the omicron variant of SARS-CoV-2 and is approved as a heterologous booster [22,23].

Self-amplifying mRNAs differ from conventional non-amplifying mRNAs in their open reading frame (ORF) region. mRNA contains five structural motifs, a 5′cap structure, a 5′ untranslated region, ORF encoding the gene of interest, a 3′ untranslated region, and a homopolymer stretch of A nucleotide. The self-amplifying mRNA ORF encodes four non-structural proteins (nsp1–4) of the virus in addition to the gene of interest. Further, the nsp1–4 polypeptide undergoes processing to generate a complex that carries replicase activity. This replicase protein helps to amplify the mRNA region encoding the gene of interest through the sub-genomic promoter that is present downstream of the nsp protein encoding ORF in the mRNA cassette. mRNA manufacturing is a cell-free process; however, it requires linearized plasmid DNA that acts as a template and a DNA-dependent RNA polymerase enzyme. Additionally, this IVT reaction also contains ribonucleotides, a few more enzymes, and cofactors. The pDNA that is used here is mostly isolated from high-cell density *E. coli* cultures; thus, there are chances that the pDNA preparation may be contaminated with genomic DNA (gDNA). As mentioned above, the pDNA acts as a template and is never consumed in the reaction, and thus shall be removed before any downstream application of the mRNA. These DNA moieties (pDNA and gDNA) are degraded enzymatically or separated using column chromatography in the presence of an appropriate matrix from the desired mRNA preparations. This separation is cumbersome as both (the desired product and DNA impurities) are chemically similar. Two other important structural motifs (cap and poly-A) are subsequently added to the mRNA chain via different approaches. A chemically modified cap structure can be added during the IVT or post-IVT steps. Alternatively, cap structures are also generated enzymatically in the presence of a capping enzyme and added post-IVT. Similarly, poly-A can be transcribed from the pDNA during the IVT or added enzymatically post mRNA synthesis. mRNA synthesis is a cell-free operation but it is very complex; thus, the identification and purity of each of the structural motifs is paramount to ensure safer and efficacious mRNA preparation. mRNA identity is mostly established by mRNA sequencing; however, the majority of sequencing chemistries are compromised for the homopolymer poly-A tail length count. mRNA purity, another critical parameter, is established by HPLC, qPCR, and spectroscopic or florescent-dye-based DNA quantitation methods. The antisense reads that may result in double-stranded RNAs are mostly checked by ELISA or dot blot methods. Undoubtedly, all these methods are robust and regulatory accepted; however, each of them has many inherent limitations, and thus, these impurities are under-quantified. 

### 3.1. mRNA Vaccine and pDNA Sequencing 

**Figure 1:**
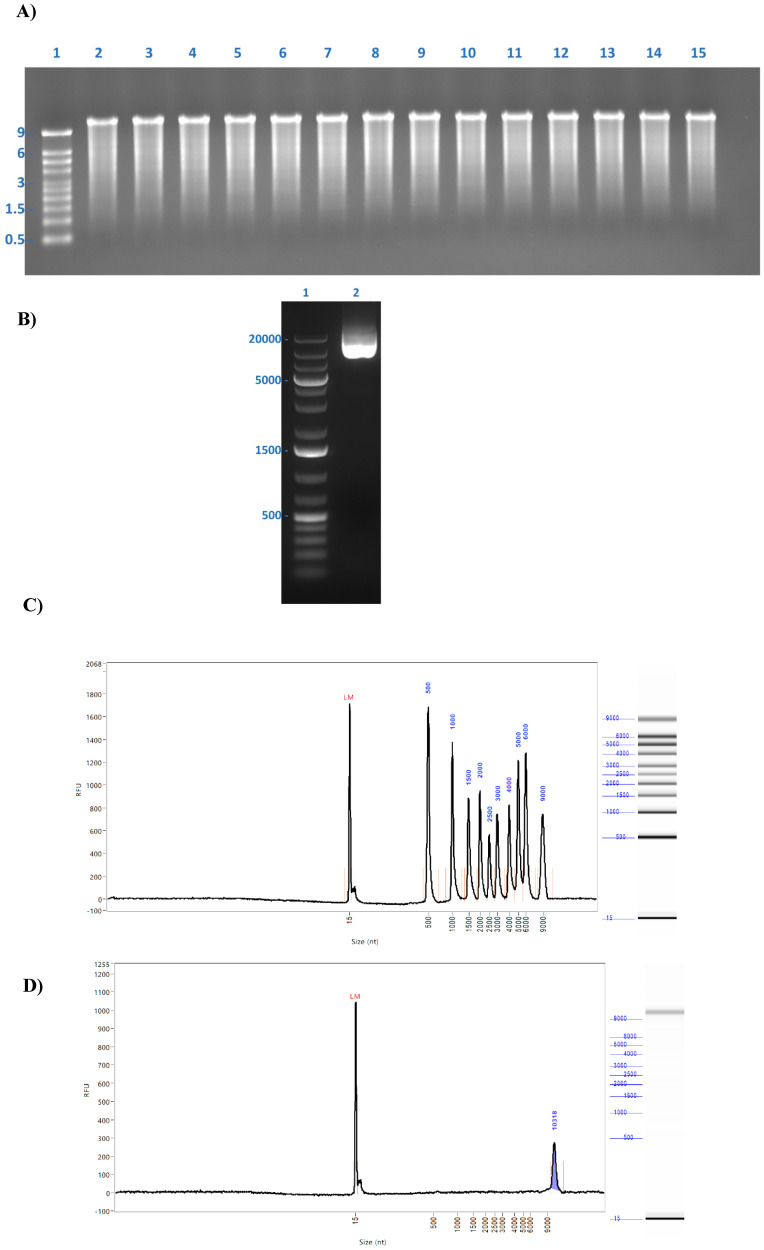
Qualitative analysis of the mRNA and DNA sample. (**A**) Ethidium bromide-stained denaturing agarose gel electrophoresis showing all 14 mRNA samples. Lane 1: Millennium™ RNA markers; lane 2: mRNA-614_E1; lane 3: mRNA-614_E2; lane 4: mRNA-614_E3; lane 5: mRNA-614_E4; lane 6: mRNA-614_E5; lane 7: mRNA-614_E6; lane 8: mRNA-614_E7; lane 9: mRNA-614_E8; lane 10: mRNA-614_E9; lane 11: mRNA-614_E10; lane 12: mRNA-628.2_E1; lane 13: mRNA-628.2_E2; lane 14: mRNA-628.2_E3; and lane 14: mRNA-628.2_E4. (**B**) Ethidium bromide-stained agarose gel electrophoresis of the pDNA samples that were processed for the Illumina NGS. Lane 1: DNA ladder; and lane 2: pDNA-628.2. (**C**) Representative image of RNA ladder on the 5200-fragment analyzer. (**D**) Representative image of one of the mRNA vaccine samples on the 5200-fragment analyzer showing a peak beyond the 9000 bases.

mRNA manufacturing was never industrialized before the COVID-19 era; thus, we manufactured numerous mRNA batches before freezing a commercially viable process for at-scale manufacturing. The mRNA quality, for the majority of the R&D and process development batches, was assessed by orthogonal approaches including NGS (MiniSeq). Illumina MiniSeq is based on a sequencing-by-synthesis approach with a fluorescently labeled reversible dye terminator technology [24,25]. Here, the optical readout of fluorescent nucleotides attached with a reversible terminator by a DNA polymerase serves as the basis for sequencing. With an error rate of less than 0.1%, Illumina’s paired-end sequencing and reversible terminator technology make it the most accurate base-by-base sequencing method available [25]. Here, the NGS data from 14 different mRNA research batches are included, wherein 10 represent mRNA-614 and the rest are mRNA-628.2. We have also considered a batch of pDNA that was used to characterize the mRNA-628.2 initial research cell banks. The mRNA was enzymatically synthesized and its quality was checked by agarose gel electrophoresis, wherein a band corresponding to approximately 12,000 bases was observed in all the samples (Figure 1A). Similarly, the pDNA sample was also verified by agarose gel electrophoresis, and the desired isoform of the plasmid was observed (Figure 1B). The mRNA integrity was checked by capillary electrophoresis on a 5200 fragment analyzer wherein a major peak was observed for the desired size, confirming the mRNA integrity (a representative picture is shown in Figure 1C,D). Next, we employed short-read sequencing methods wherein millions of sequencing reactions take place in a massively parallel manner. Short-read sequencing provides high accuracy as compared to long-read sequencing [25]. Library preparation, sequencing, and data analysis are the steps included as a part of the NGS workflow [26]. Here, the first strand of DNA was chemically synthesized, followed by the second strand, and finally, it was fragmented and amplified. The amplified product in the form of a library was checked on the fragment analyzer for the median size distribution. Since we used paired-end 250 bp chemistry on the Illumina MiniSeq platform, we targeted the size of 150–500 bp for our adaptor-ligated and amplified library. Similarly, the plasmid DNA was also processed as detailed in the Methods section, and the fragmented amplified library was prepared. All these libraries were normalized and loaded into the sequencing cartridge and sequenced on the Illumina platform. These samples were run at different time intervals, and we have pulled the data of the desired samples for workflow demonstration purposes. For the majority of samples, the cluster passing filter varied from 65 to 97% with a read-1 Q30 passing filter of up to 99% and a read-2 Q30 passing filter of up to 98%. The average cluster density for the run was 282 K/mm^2^. 

### 3.2. Identity and Purity Analysis 

**Figure 2:**
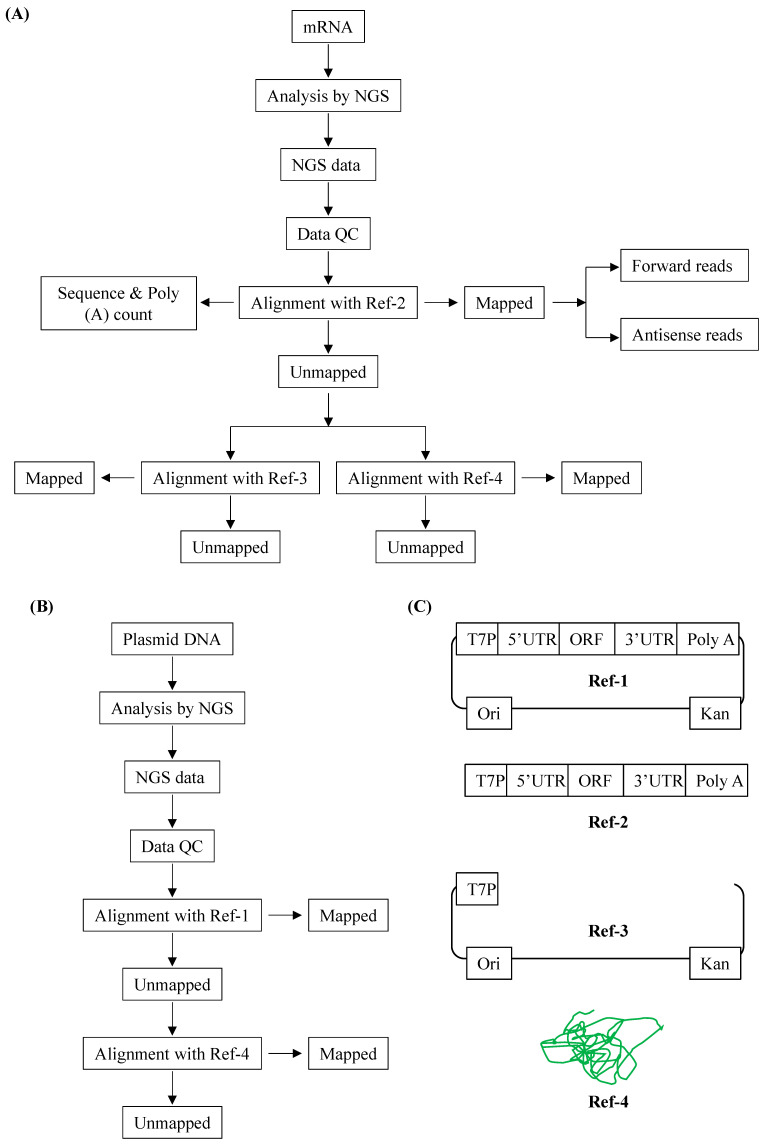
Proposed process flow for the NGS data analysis and pictorial representation of reference files. (**A**) Process flow of NGS data analysis of mRNA. Red boxes represent the contaminating reads. (**B**) Process flow of NGS data analysis of plasmid DNA. Red boxes represent the contaminating reads. (**C**) Ref-1—complete circular plasmid sequence that was used for the mRNA manufacturing; Ref-2—sequence of the mRNA replicon; Ref-3—Ref-1 minus Ref-2 depicting the backbone sequences; and Ref-4—*E. coli* DH5α sequence.

The entire NGS data analysis was segregated into four sequential phases for mRNA (Figure 2A) and two for plasmid DNA analysis (Figure 2B). We created four different reference files: Ref-1 containing the complete pDNA sequence (Ref-1_614 and Ref-1_628.2), Ref-2 containing the sequences that will be transcribed into the mRNA replicon (Ref-2_614 and Ref-2_628.2), Ref-3 containing the Ref-1 minus Ref-2 sequences (identical for 614 and 628.2), and Ref-4 containing the genomic DNA (gDNA) of *E. coli* DH5α (identical for 614 and 628.2) (Figure 2C). 

For mRNA, the first phase involves the confirmation of the mRNA replicon sequence with the reference file (Ref-2_614 and Ref-2_628.2) that includes the poly-A length as well. The next two phases primarily focus on identifying the nucleotide impurities of pDNA and gDNA. The second phase evaluates the pDNA impurity using the unmapped reads from the first phase followed by its alignment with Ref-3. The third phase assesses the gDNA impurity using the unmapped reads of the second phase following its alignment with Ref-4. Lastly, the fourth phase checks for the antisense reads that are generated as a by-product of in vitro transcription (IVT) using all the NGS data. 

The raw data of all the NGS batches were pre-processed using the Fastqc and Trimmomatic-0.39 tools. In the first phase of the analysis, the pre-processed reads of mRNA were mapped with Ref-2 for their respective variants (614 with Ref-2_614 and 628.2 with Ref-2_628.2) using the Bowtie2 tool. The resultant SAM files were used to generate the sorted and indexed bam files which were then visualized using Integrated Genomics Viewer (IGV). The IGV plots of alignments are provided in Supplementary File S1, wherein two tracks viz. coverage and alignments are represented. The number of reads at each nucleotide position is indicated by the coverage track, whereas gray bars in the lower alignments track denote distinct, individual alignments and the color of the bars indicates similarity found from the alignment with the reference files. For all the mRNA and pDNA samples, significant sequence coverage and similarity were observed with the respective reference files viz. Ref-1_628.2, Ref-2_614, and Ref-2_628.2. The overall mapping percentage for all the reads (R1 and R2) with Ref-1 and Ref-2 files was 89 to ~99% (refer to Supplementary File S2 for details). This confirms the sequence identity of all the samples considered in the present study.

**Figure 3:**
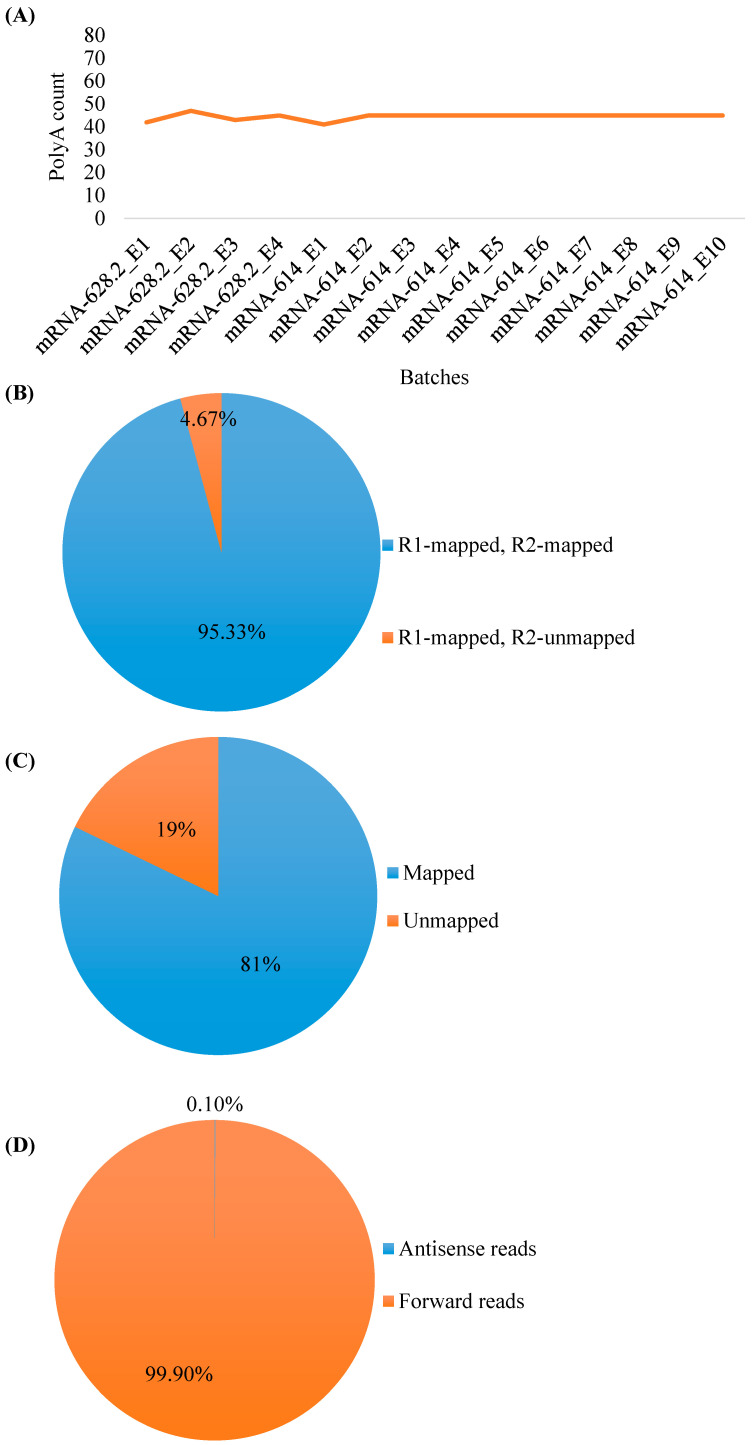
Analysis of NGS data for mRNA quality assessment of 14 batches. (**A**) Poly(A) count for all 14 batches obtained by exploring aligned reads in the IGV. (**B**) Pie-chart showing proportion of NGS reads aligned with the reference genome (Ref-2). The reads are divided into four different categories: (i) both forward (R1) and reverse reads (R2) are mapped, (ii) R1 are mapped and R2 are unmapped, (iii) R1 are unmapped and R2 are mapped, and (iv) both R1 and R2 are unmapped. (**C**) Pie-chart showing the proportion of unmapped reads of the NGS data of 14 batches. The remaining portion of the pie-chart represents mapped reads. (**D**) Pie-chart showing the composition of off-target RNA contamination in 14 batches represented by antisense reads. The remaining portion of the pie-chart represents forward reads.

Further, after exploring the aligned reads in the IGV, the poly-A tail length was estimated in the range of 41–47 (Figure 3A) for all the mRNA batches. In the second phase, the aligned reads were segregated into four different categories viz. (i) both forward (R1) and reverse reads (R2) are mapped, (ii) R1 are mapped and R2 are unmapped, (iii) R1 are unmapped and R2 are mapped, and (iv) both R1 and R2 are unmapped. Next, all the unmapped reads from the previously described four categories were extracted, wherein categories 3 and 4 did not represent any reads. The first category contained our desirable data that represent an average of 95.33% reads of entire NGS data from all 14 mRNA batches. The remaining 4.67% of the reads corresponded to category 2 (Figure 3B). Further slicing of category 2 (4.67% reads) revealed that an average of 19% of reads were unmapped (Figure 3C). These unmapped reads were our focus as they represented the contamination. Upon alignment with Ref-3, we did not find any significant similarity. Later, in the third phase, we aligned the unmapped reads with Ref-4, wherein, again, no significant similarity was found. The possible interpretation of the presence of these reads could be connected with the NGS library preparation step, e.g., in PCR amplification, or an error may occur in the ligation of adapters, or there may be variations in the shearing of RNA that sometimes generate a population without adaptors. Another reason would be RNA sequencing artifacts such as errors in extracting RNA from tissues/cells. Moreover, as sequencers do not possess 100% accuracy, their error rate may also result in sequencing artifacts. In the fourth phase, for the off-target RNA identification, after employing SAM tools to separate mapped reads that originated from the forward and reverse strands of Ref-1, only 0.1% antisense reads representing off-target RNA were detected (Figure 3D) in the tested mRNA batches, and this information was used later to optimize our IVT processes. The entire NGS data statistics is summarized in Supplementary File S2, and IGV plots depicting antisense reads are demonstrated in Supplementary File S3, wherein antisense reads track, forward reads track, and sequence coverage track were signified. For all 14 mRNA samples, the lowest percentage, i.e., ~0.1%, was observed for the antisense reads, whereas the maximum percentage, i.e., ~99% (Supplementary File S2), was observed for the forward reads, confirming minimal content of off-target RNA in the samples. It is noteworthy to mention that the quality landscape of mRNA vaccines is continuously evolving based on the incremental datasets published by researchers. The current guidance of DNA impurities in mRNA vaccines from the EMA states a limit of 330 ng DNA/mg RNA [27], and from the US FDA, a limit of less than 10 ng DNA/dose of RNA [28]. The DNA impurities from the mRNA samples are removed using column chromatography or enzymatic DNA degradation. 

For pDNA analysis, the first phase involves the pDNA identity by aligning total reads with Ref-1. The second phase evaluates the gDNA contamination by aligning unmapped reads of the first phase with Ref-4. In the first phase, we first mapped our pDNA data with Ref-1_628.2. Here, 98% of reads represent Category 1; further segregation of the remaining 2% of reads corresponding to Category 2 revealed that 20% of them are entirely unmapped (Supplementary File S2). During the second-phase analysis, the unmapped reads were further mapped on the Ref-4. The data confirm 7% query coverage and identity of 100%. This specifies the detected gDNA contamination in the pDNA sequencing data. Here, we used one of the commercially available miniprep kits that did not eliminate the gDNA in the pDNA preparation. 

In a similar study by Helen et al. [11], they proposed a simplified protocol known as VAX-seq for the quality assessment of mRNA vaccines, including length, integrity, sequence, and purity. To facilitate VAX-seq, they also created the software toolkit Mana (https://github.com/scchess/Mana, accessed on 10 November 2024), which offers comprehensive and automated reports on mRNA quality that can be routinely generated. Mana works by taking aligned NGS libraries as input and generates reports that are composed of sequence identity, purity, and mRNA length. They analyzed mRNA vaccines with VAX-seq by performing long-read nanopore cDNA sequencing and determined the crucial parameters for mRNA quality viz. length of the 3′-poly(A) tail, sequence identity, integrity, and DNA and RNA contamination. Moreover, they designed and produced a reference eGFP mRNA to demonstrate the usage of VAX-seq, thereby validating their protocol. 

## 4. Conclusions

The COVID-19 wave reversal by mRNA vaccines has provided momentum to the technology, and now, it is also being evaluated for various other indications. Recently, a mRNA vaccine for Respiratory Syncytial Virus from Moderna was also approved by the US FDA [29]. Here, we substantiated the advantages of short-RNA sequencing methods that hold promise for the quality control of mRNA vaccines. Furthermore, we are also proposing the adaptation of incremental NGS data to identify nucleotide-based impurities in NAP. The product development guidance document shall recommend NGS technology as it provides tangible quality control outcomes for nucleic acid-based impurities. Such a synergistic orthogonal quality control approach will ensure the delivery of quality-by-design products to the end users or patients. We also suggest the following BEST practices/focus areas for the next couple of years to convert the “nucleic acid platform” into a modality: Biopharma’s vision is to promote collaborations among the stakeholders for building a nucleic acid ecosystem; Efficacy and safety awareness programs for the successful deployment of nucleic acid-based products; Service providers training and supportive infrastructures to address the global disparity for its deployment; Treatment cost to make it available for Low- and Middle-Income Countries (LMICs) as well by employing cutting-edge innovations in the manufacturing processes.

## Data Availability

The original contributions presented in the study are included in the article/Supplementary Material, NGS data are available on request from the corresponding author.

## Supplementary Materials

The following supporting information can be downloaded at https://www.mdpi.com/article/10.3390/pharmaceutics17010030/s1: Supplementary File S1: IGV plots of complete alignments of mRNA/DNA; Supplementary File S2: Statistical analysis of NGS reads of mRNA/DNA; Supplementary File S3: IGV plots of alignments for antisense reads of mRNA.
