# Supplementary material for: Leveraging Next-Generation Sequencing Application from Identity to Purity Profiling of Nucleic Acid-Based Products
Source: Pharmaceutics. 2024 Dec 28;17(1):30. doi: 10.3390/pharmaceutics17010030 (PMC11769349; doi:10.3390/pharmaceutics17010030)
Supplement: Supplementary file 1 [file pharmaceutics-17-00030-s001.zip › S1.pdf]

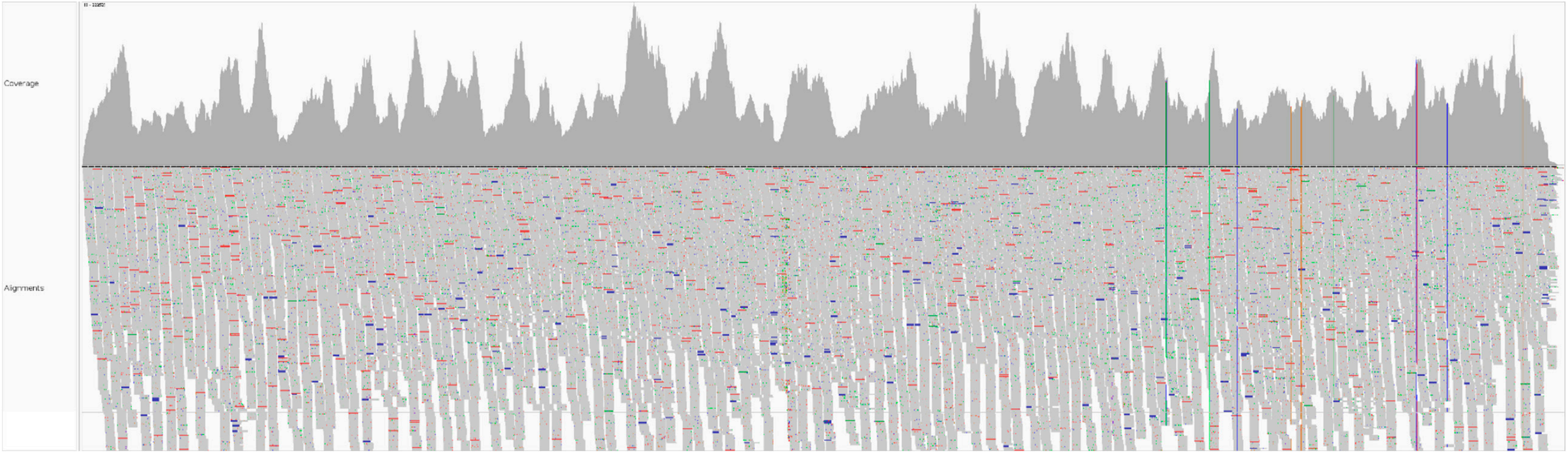

mRNA-614_E1

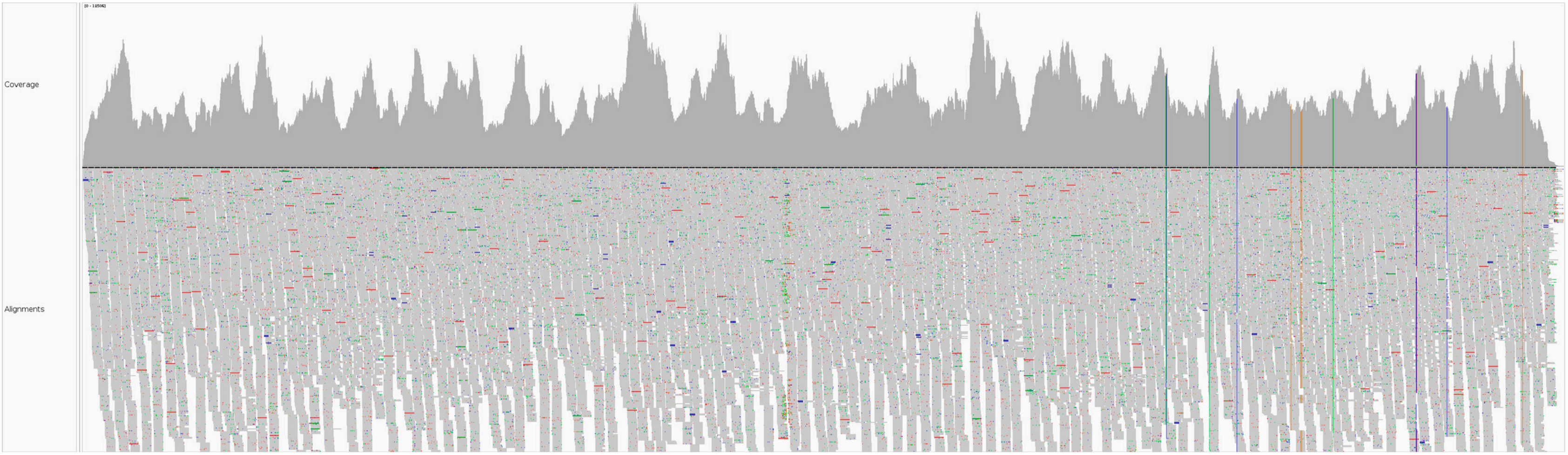

mRNA-614_E2

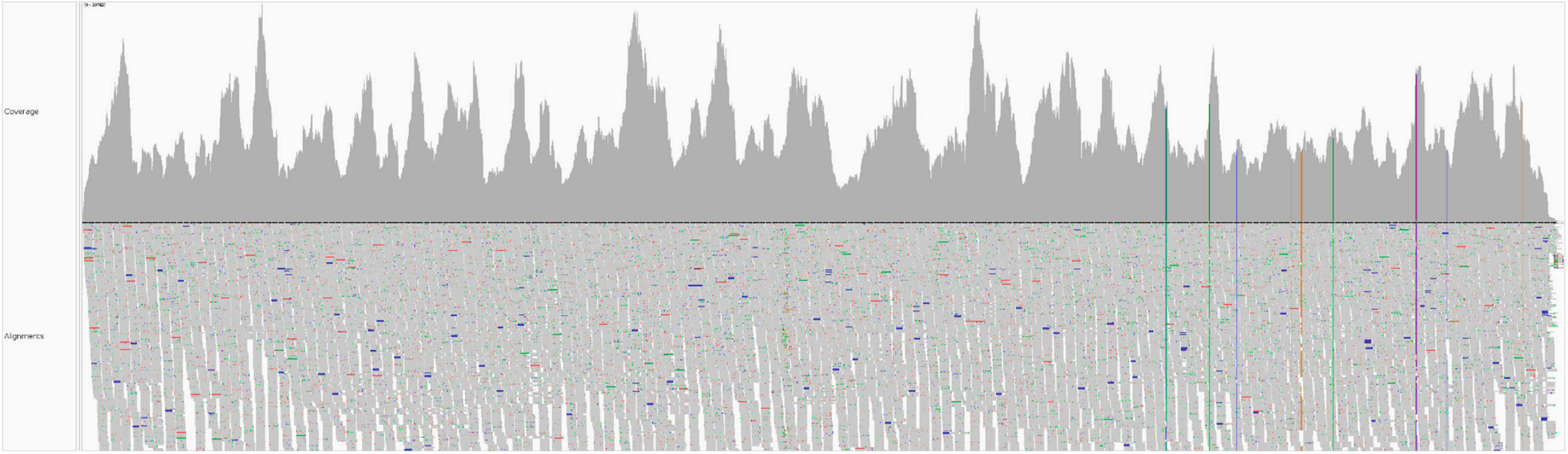

mRNA-614_E3

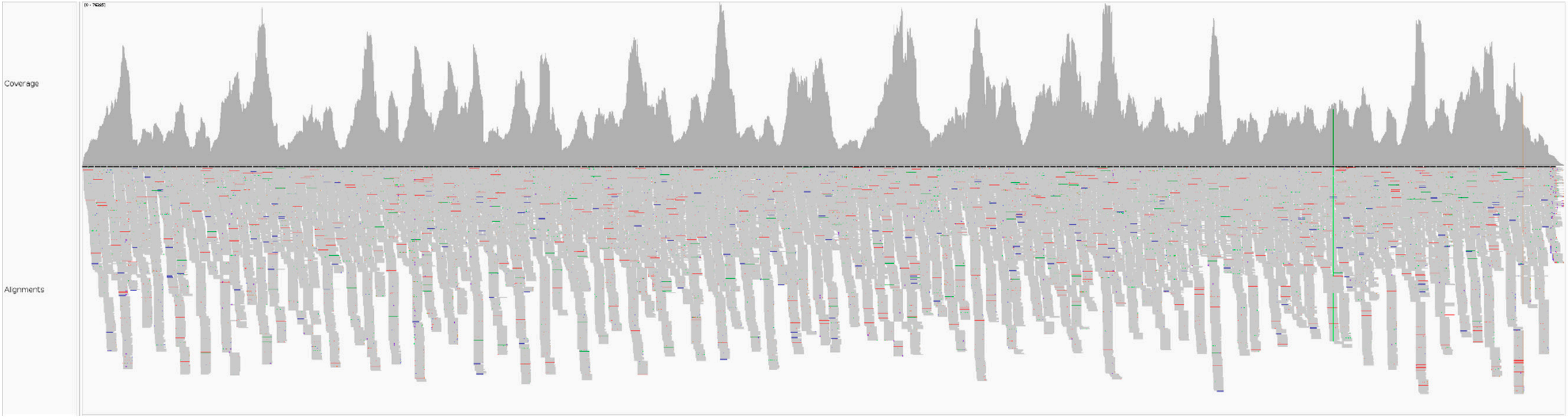

mRNA-614_E4

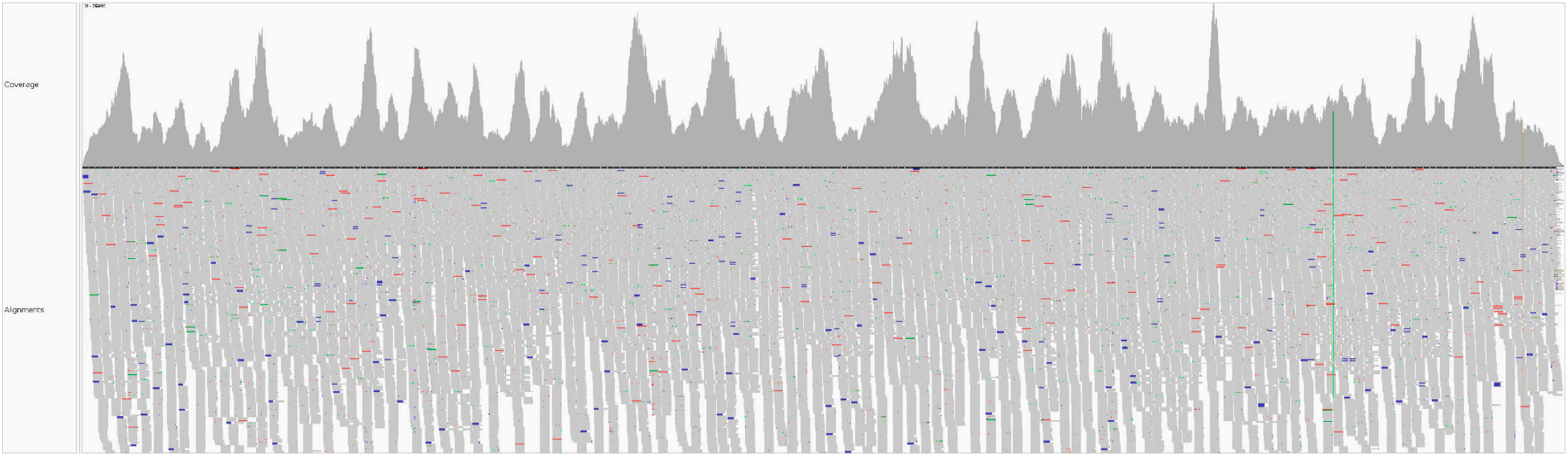

mRNA-614_E5

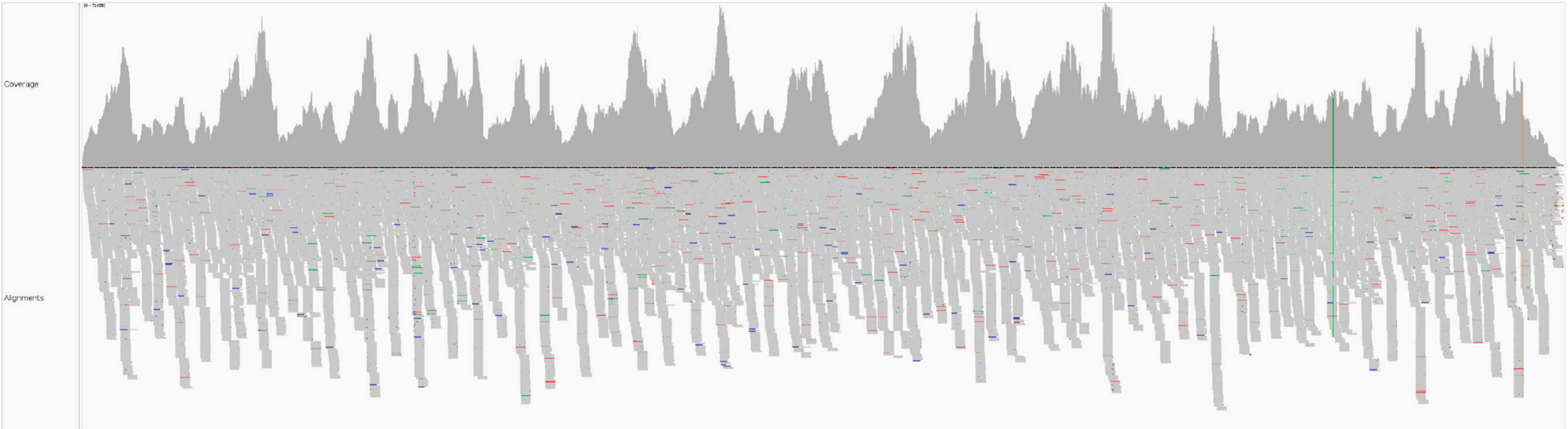

mRNA-614_E6

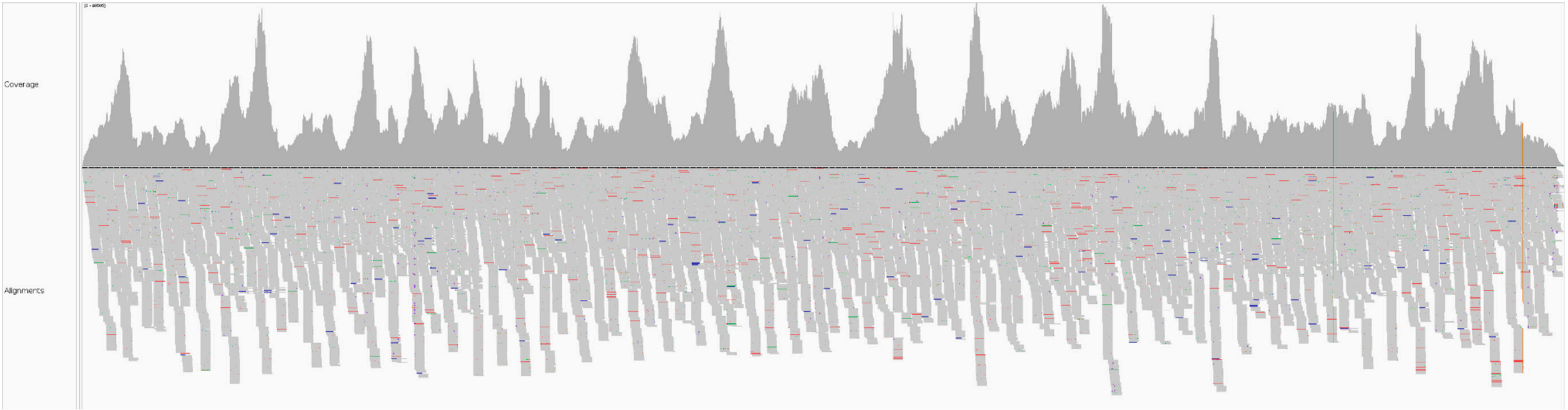

mRNA-614_E7

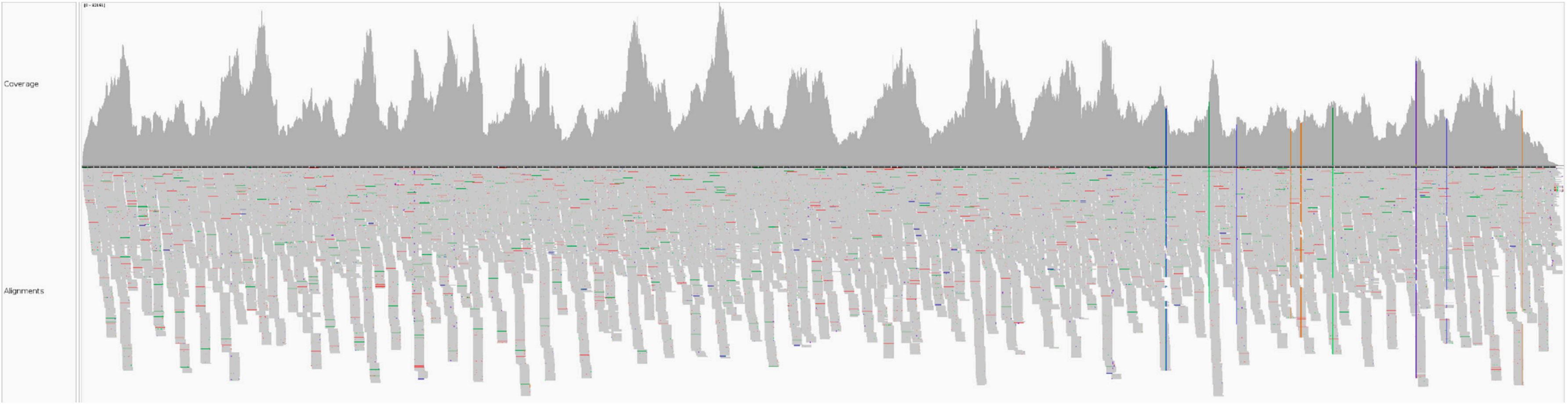

mRNA-614_E8

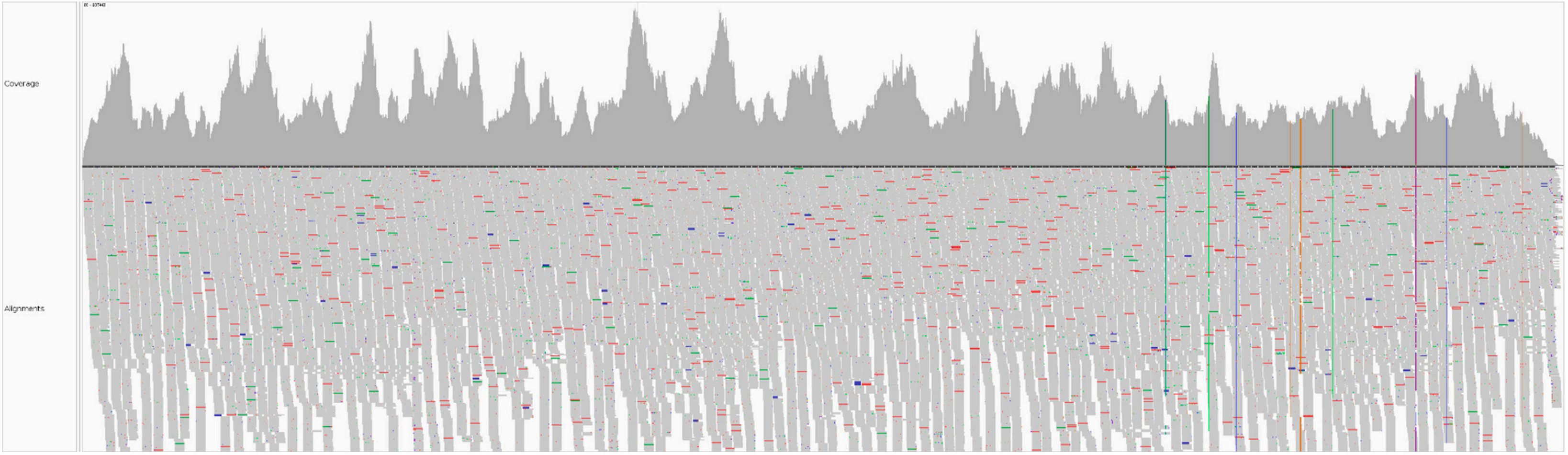

mRNA-614_E9

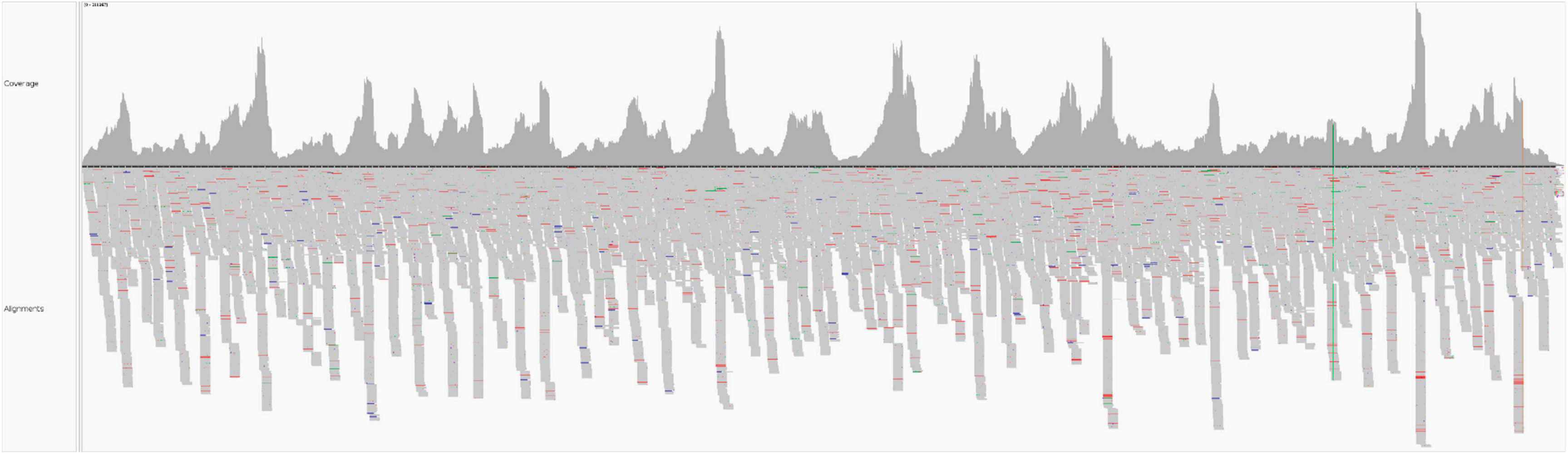

mRNA-614_E10

Figure. S1.1: NGS reads of mRNA-614 samples aligned with Ref-2_614 are represented in the IGV.

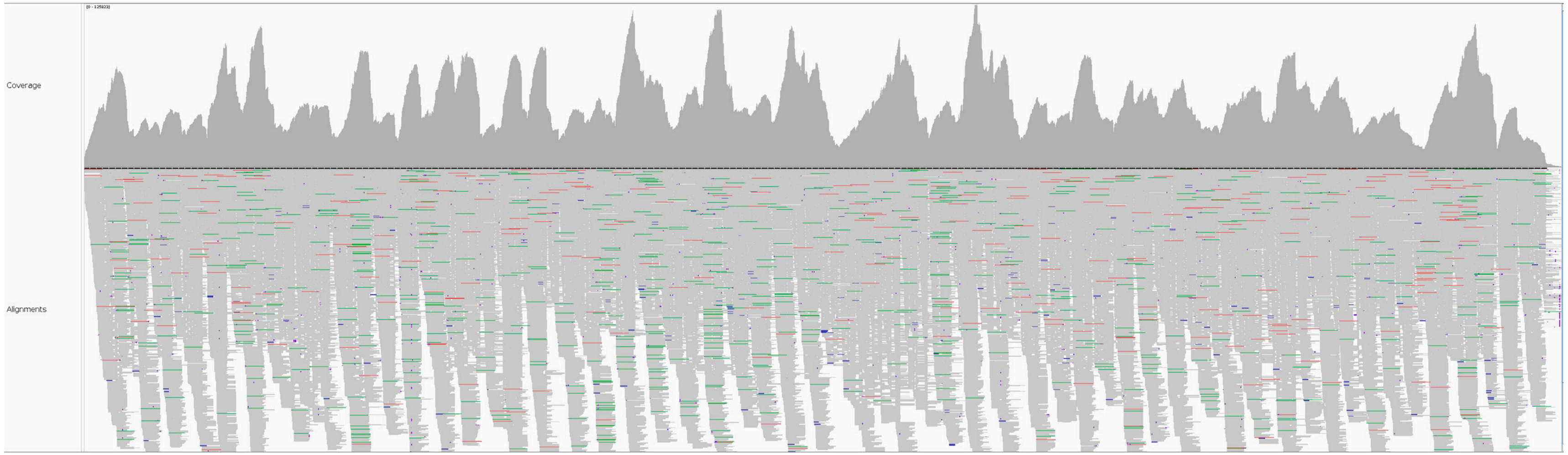

mRNA-628.2_E1

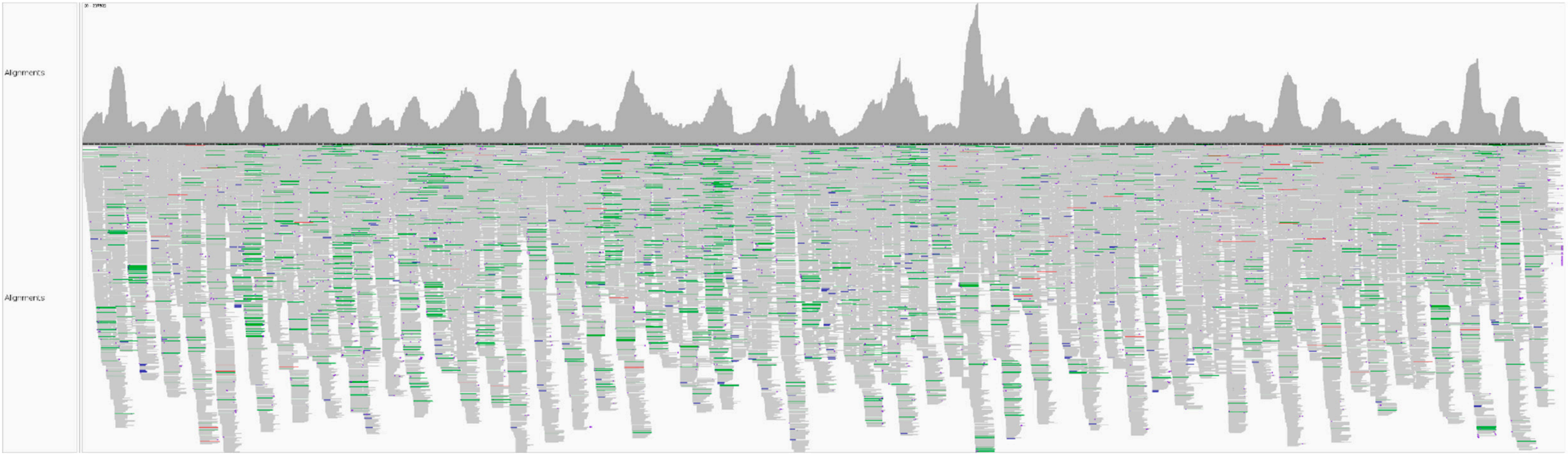

mRNA-628.2_E2

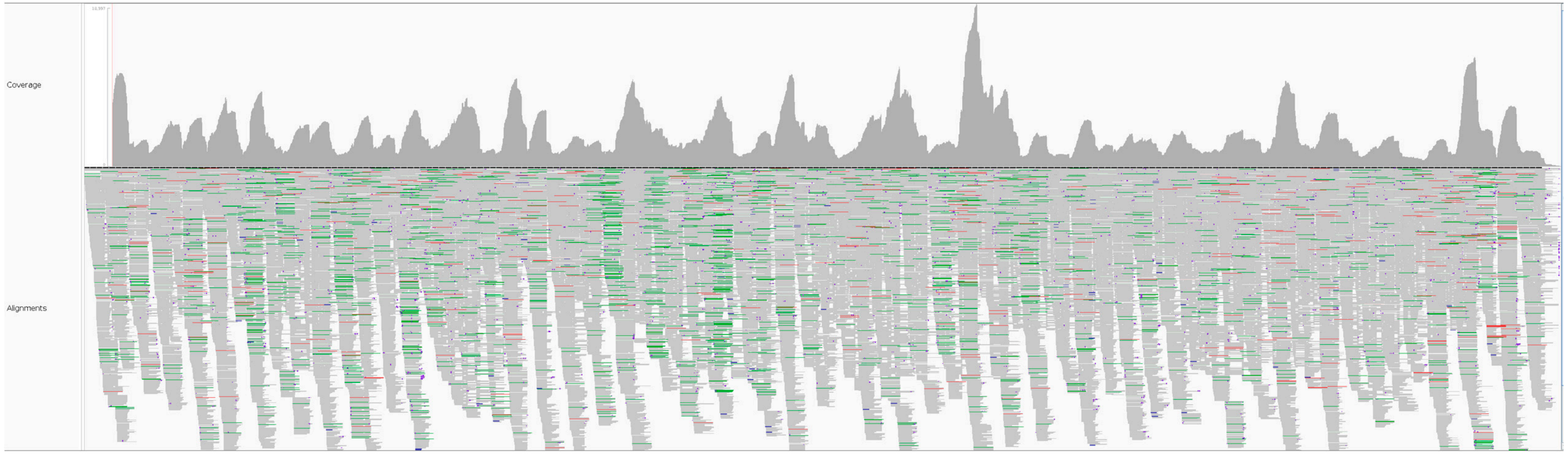

mRNA-628.2_E3

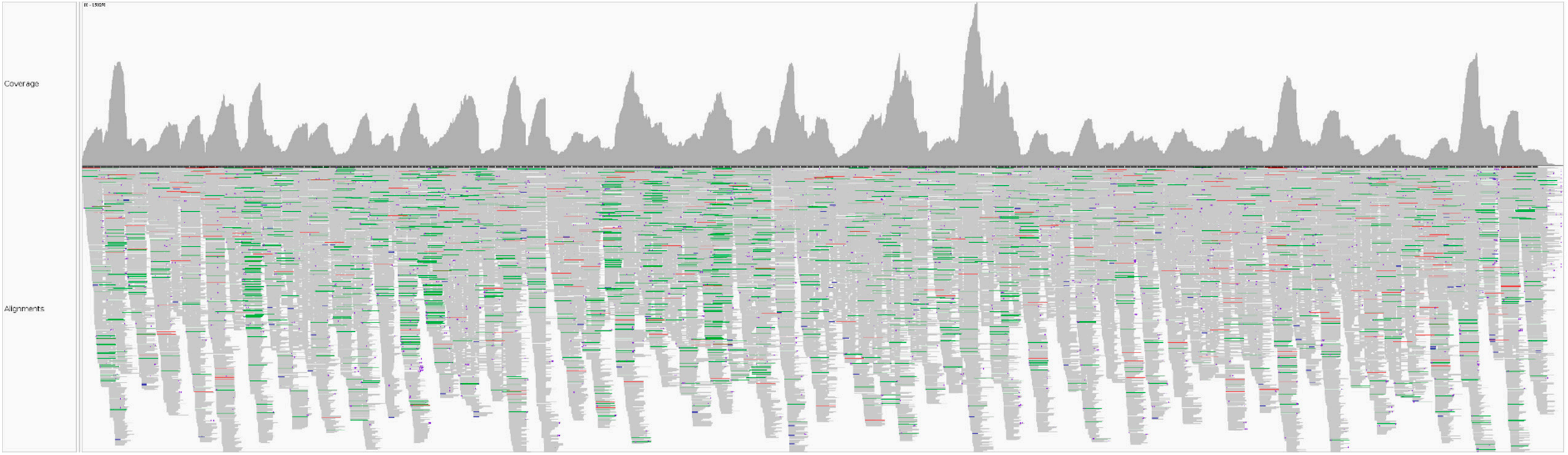

mRNA-628.2_E4

Figure S1.2: NGS reads of mRNA-628.2 samples aligned with Ref-2_628.2 are represented in the IGV.

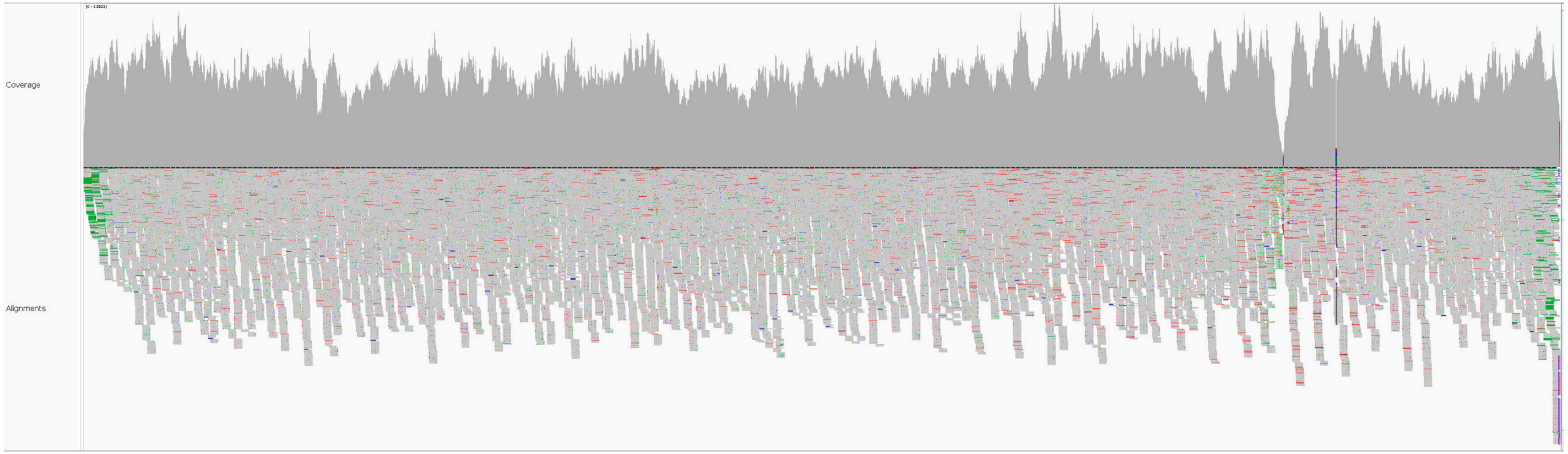

Figure S1.3: NGS reads of the pDNA-628.2 sample aligned with Ref-1_628.2 are represented in the IGV.
