# Supplementary material for: Leveraging Next-Generation Sequencing Application from Identity to Purity Profiling of Nucleic Acid-Based Products
Source: Pharmaceutics. 2024 Dec 28;17(1):30. doi: 10.3390/pharmaceutics17010030 (PMC11769349; doi:10.3390/pharmaceutics17010030)
Supplement: Supplementary file 1 [file pharmaceutics-17-00030-s001.zip › S3.pdf]

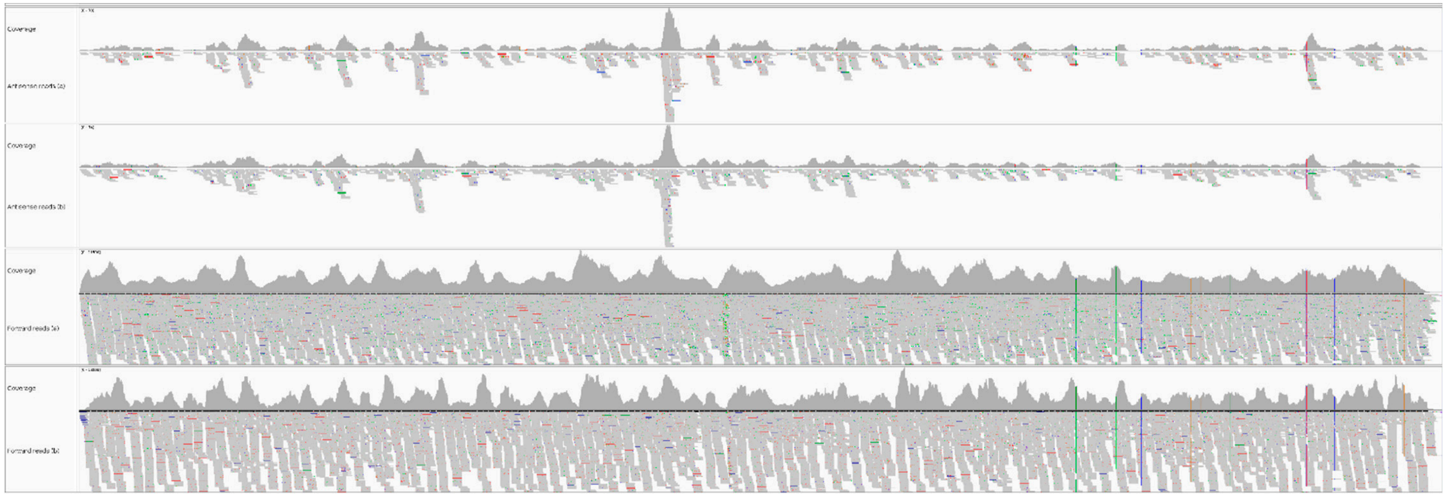

mRNA-614_E1

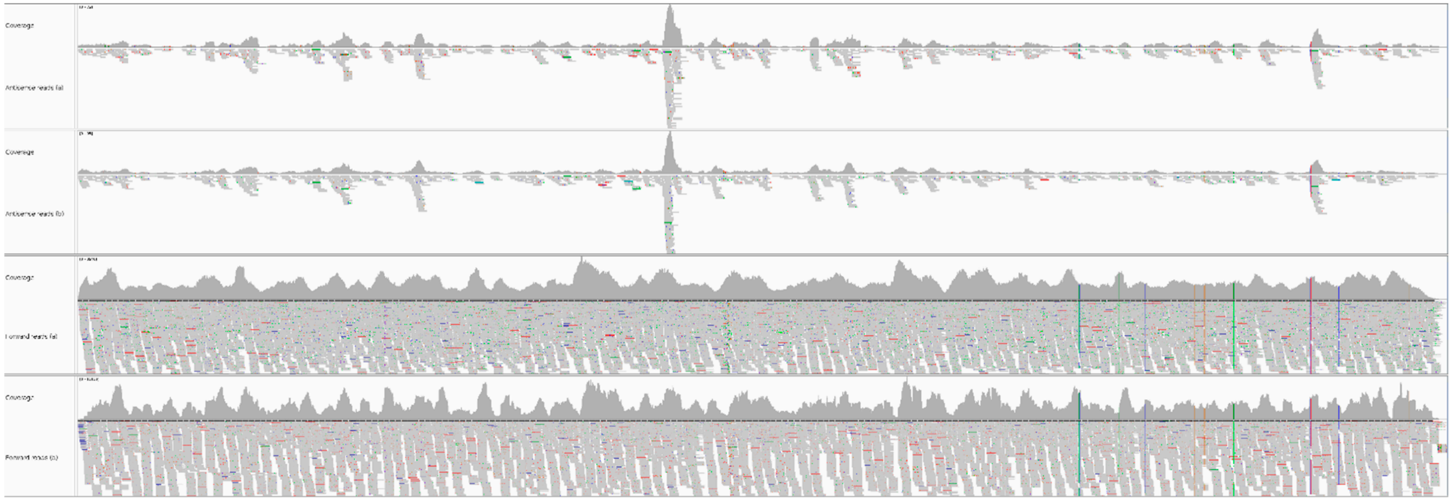

mRNA-614_E2

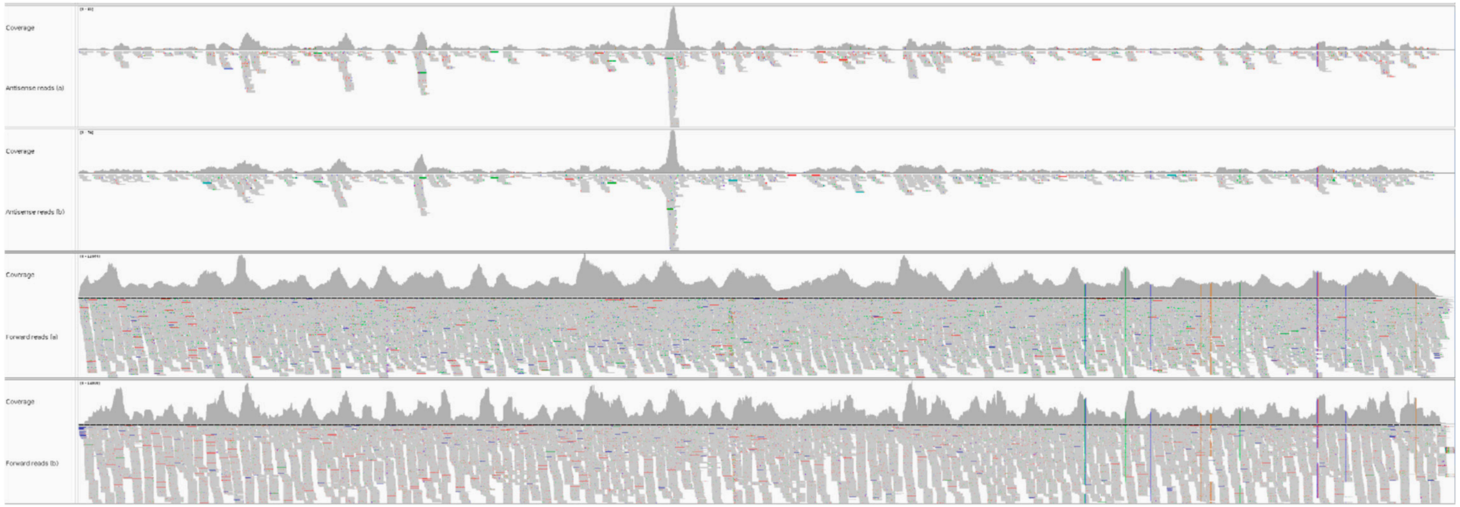

mRNA-614_E3

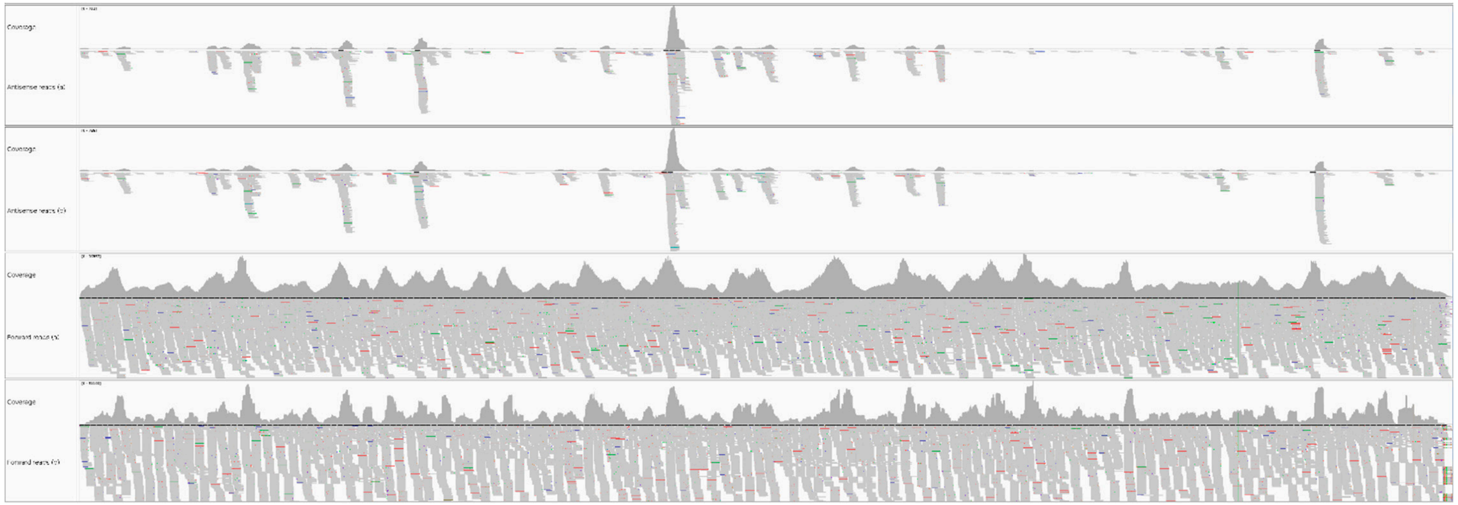

mRNA-614_E4

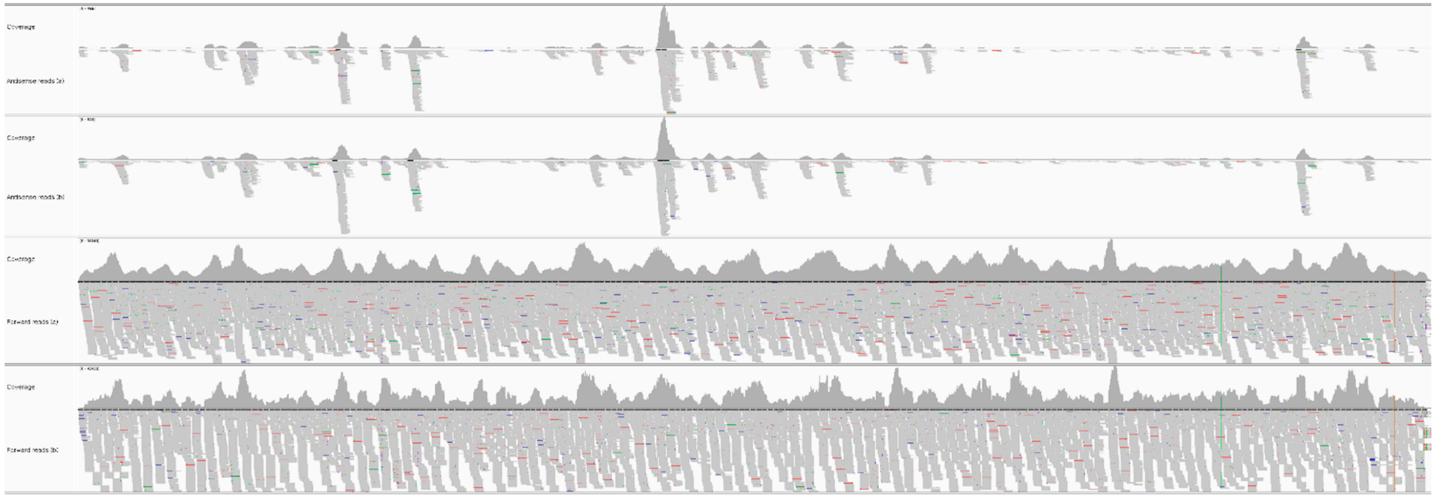

mRNA-614_E5

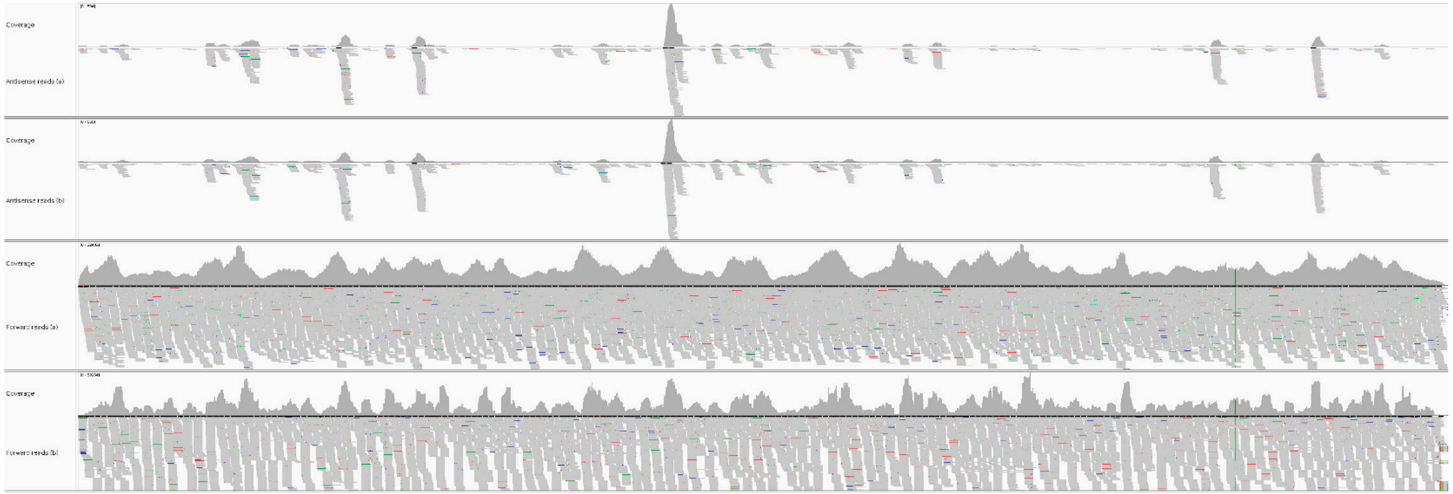

mRNA-614_E6

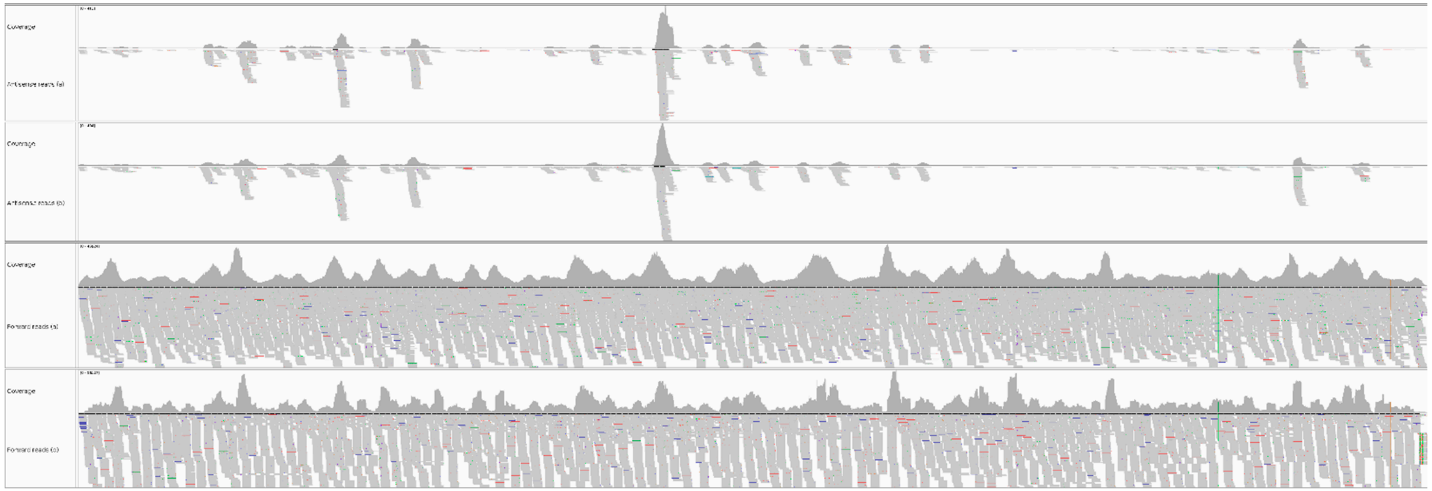

mRNA-614_E7

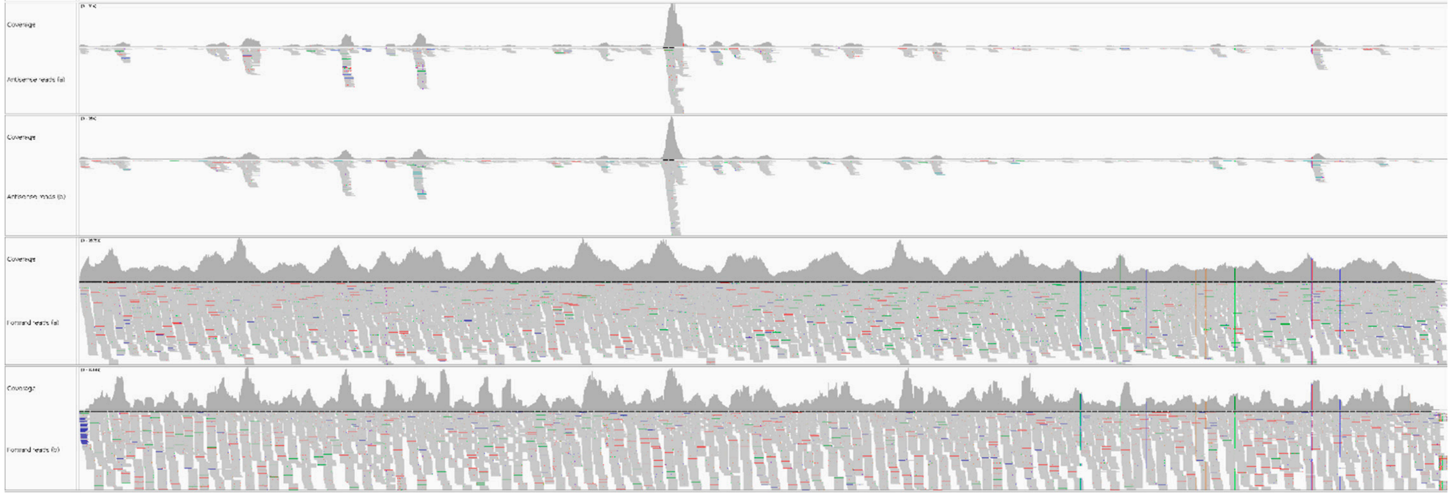

mRNA-614_E8

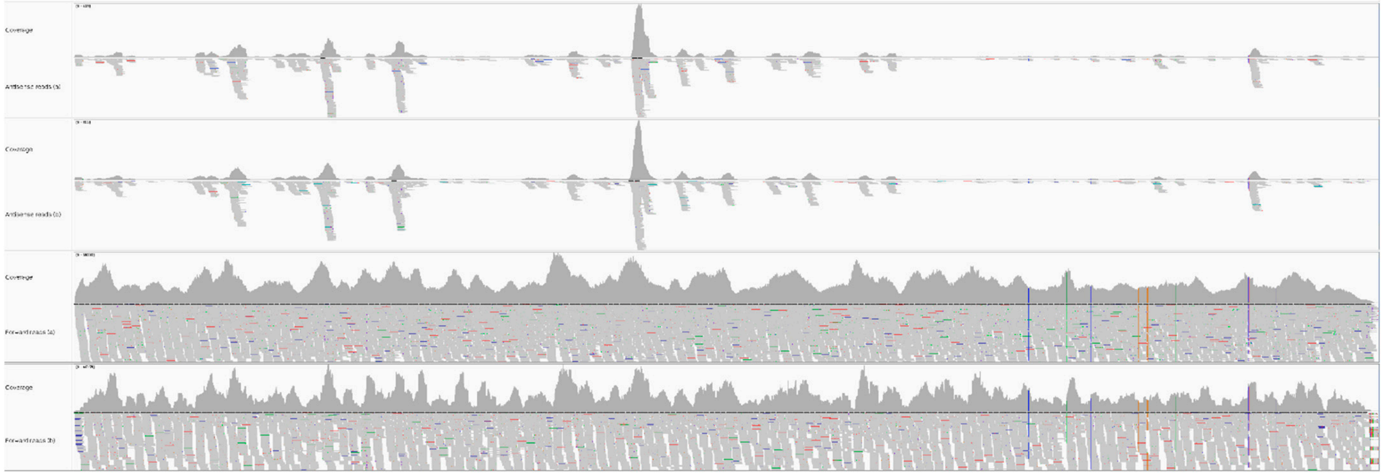

mRNA-614_E9

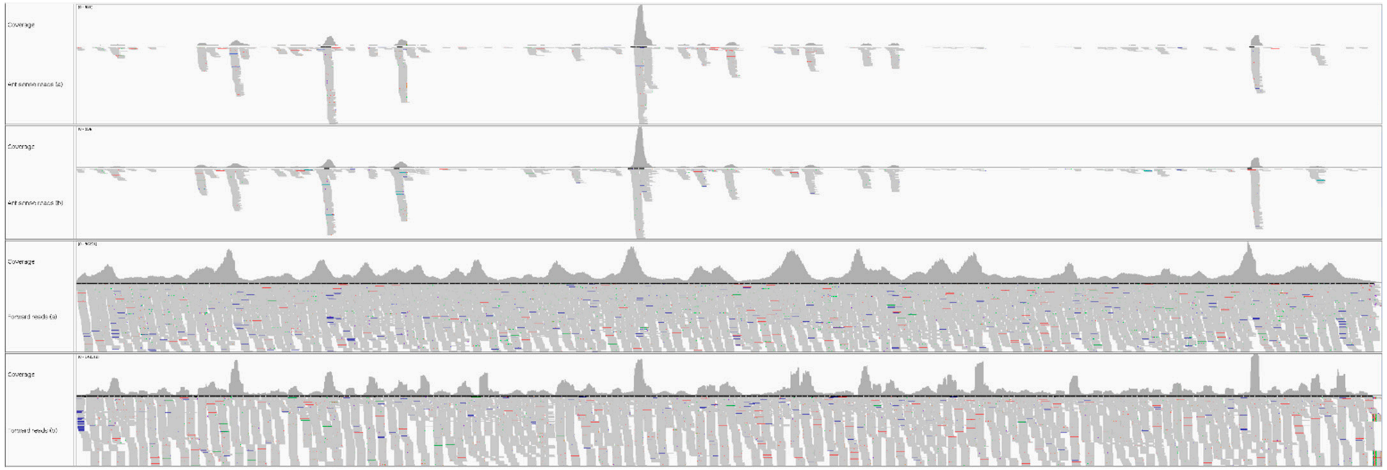

mRNA-614_E10

Figure S3.1: IGV view of aligned mRNA-614 sample reads with Ref-2_614 segregated into antisense and forward reads.

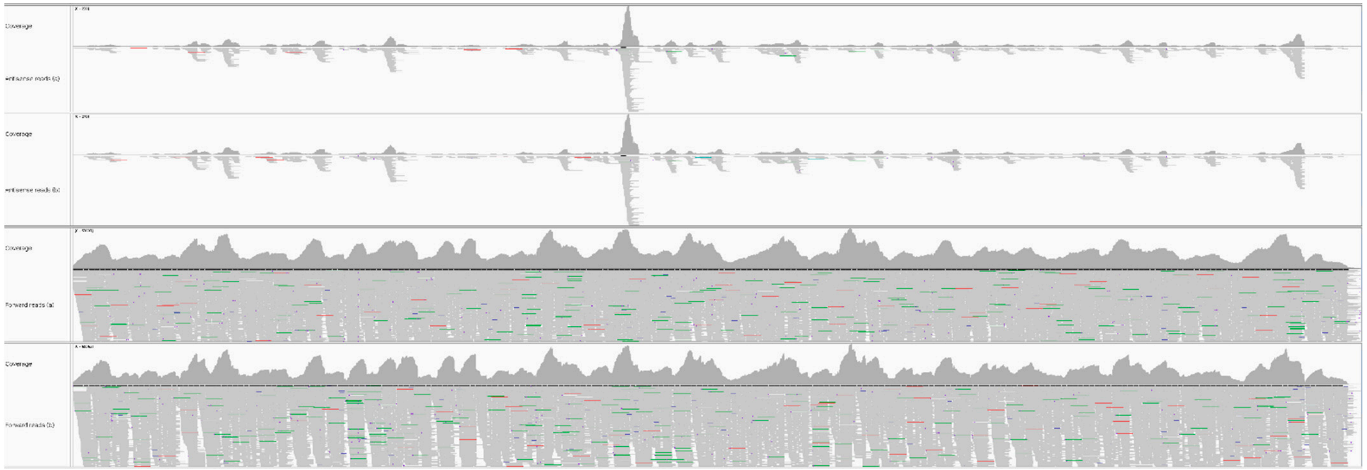

mRNA-628.2_E1

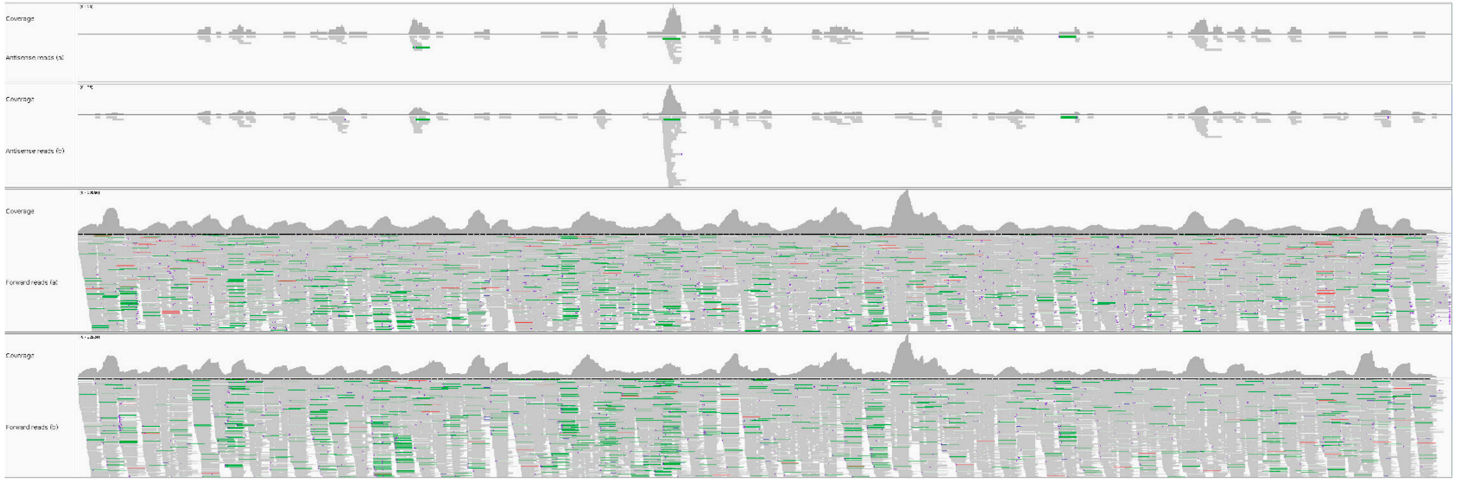

mRNA-628.2_E2

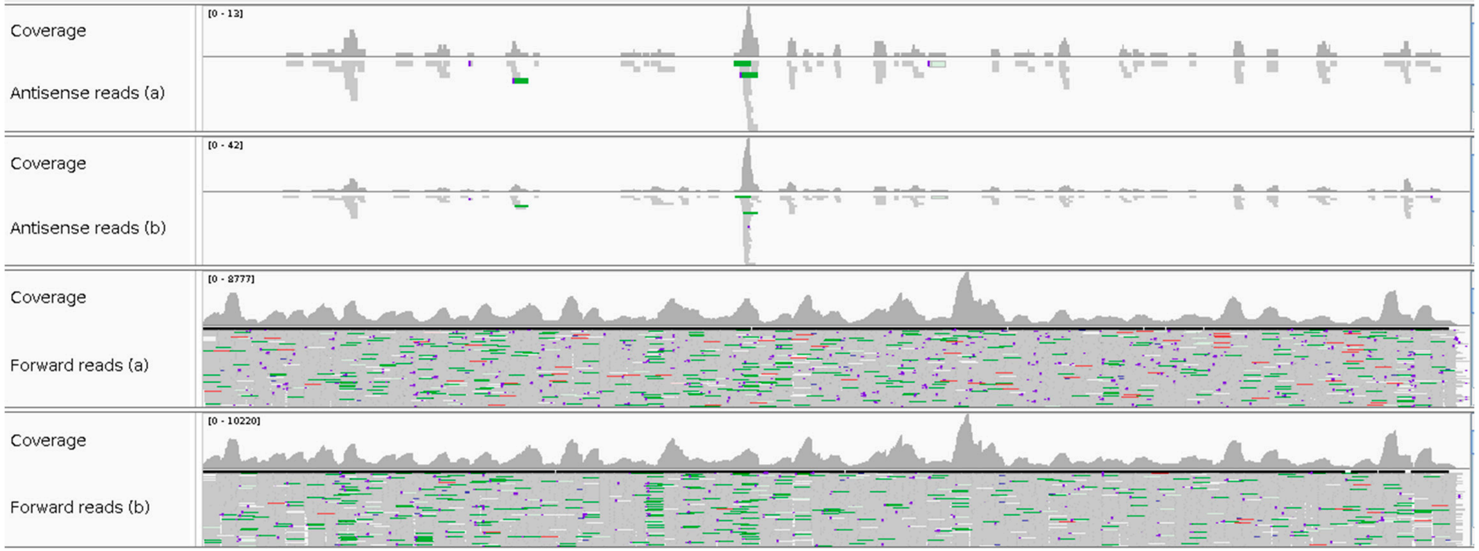
Coverage
Antisense reads (a)
[0 - 13]
Coverage
Antisense reads (b)
[0 - 42]
Coverage
Forward reads (a)
[0 - 8777]
Coverage
Forward reads (b)
[0 - 10220]

mRNA-628.2_E3

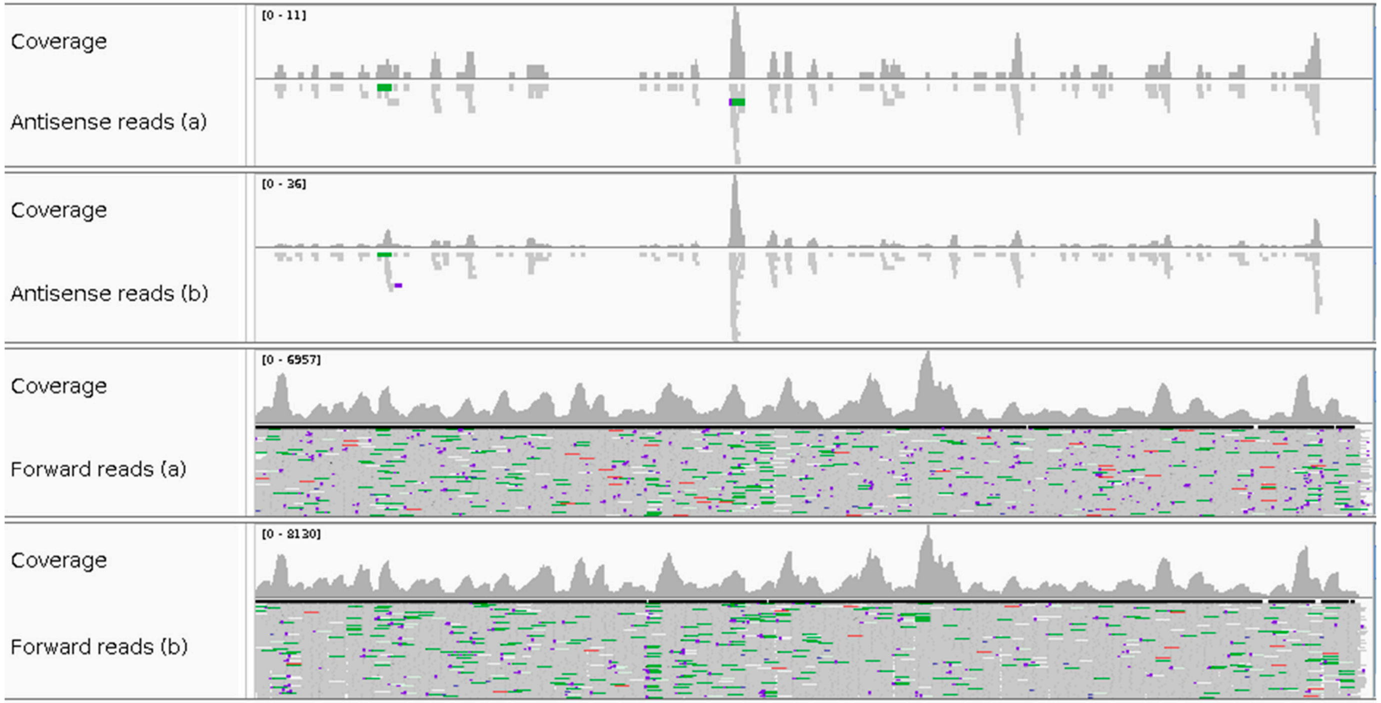

mRNA-628.2_E4

Figure S3.2: IGV view of aligned mRNA-628.2 sample reads with Ref-2_628.2 segregated into antisense and forward reads.
